# Real‐world glycaemic outcomes in patients with type 1 diabetes using glucose sensors—Experience from a single centre in Dublin

**DOI:** 10.1002/edm2.469

**Published:** 2024-01-17

**Authors:** Robert E. Lyons, Roshaida Abdul Wahab, Sue Yee Goh, Cathy Breen, Amanda Rhynehart, Mary O'Scannail, Hannah Jade Kelly, Karl Neff, Donal O'Shea, Ronan Canavan, Wan Aizad Wan Mahmood

**Affiliations:** ^1^ Endocrinology and Diabetes Unit St. Columcille's Hospital Dublin Ireland; ^2^ Diabetes Complications Research Centre, Conway Institute University College Dublin Dublin Ireland; ^3^ School of Medicine University College Dublin Dublin Ireland

**Keywords:** continuous glucose monitor, DAFNE, Dexcom G6, flash glucose monitor, FreeStyle Libre, Guardian 3, type 1 diabetes

## Abstract

**Aims:**

To evaluate changes in glycated haemoglobin (HbA_1_c) and sensor‐based glycaemic metrics after glucose sensor commencement in adults with T1D.

**Methods:**

We performed a retrospective observational single‐centre study on HbA_1_c, and sensor‐based glycaemic data following the initiation of continuous glucose monitoring (CGM) in adults with T1D (*n* = 209).

**Results:**

We observed an overall improvement in HbA_1_c from 66 (59–78) mmol/mol [8.2 (7.5–9.3)%] pre‐sensor to 60 (53–71) mmol/mol [7.6 (7.0–8.6)%] on‐sensor (*p* < .001). The pre‐sensor HbA_1_c improved from 66 (57–74) mmol/mol [8.2 (7.4–8.9)%] to 62 (54–71) mmol/mol [7.8 (7.1–8.7)%] within the first year of usage to 60 (53–69) mmol/mol [7.6 (7.0–8.4)%] in the following year (*n* = 121, *p* < .001). RT‐CGM‐user had a significant improvement in HbA_1_c (Dexcom G6; *p* < .001, *r* = 0.33 and Guardian 3; *p* < .001, *r* = 0.59) while a non‐significant reduction was seen in FGM‐user (Libre 1; *p* = .279). Both MDI (*p* < .001, *r* = 0.33) and CSII group (*p* < .001, *r* = 0.41) also demonstrated significant HbA_1_c improvement. Patients with pre‐sensor HbA_1_c of ≥64 mmol/mol [8.0%] (*n* = 125), had attenuation of pre‐sensor HbA_1_c from 75 (68–83) mmol/mol [9.0 (8.4–9.7)%] to 67 (59–75) mmol/mol [8.2 (7.6–9.0)%] (*p* < .001, *r* = 0.44). Altogether, 25.8% of patients achieved the recommended HbA_1_c goal of ≤53 mmol/mol and 16.7% attained the recommended ≥70% time in range (3.9–10.0 mmol/L).

**Conclusions:**

Our study demonstrated that minimally invasive glucose sensor technology in adults with T1D is associated with improvement in glycaemic outcomes. However, despite significant improvements in HbA_1_c, achieving the recommended goals for all glycaemic metrics remained challenging.

## Graphical abstract

Use of minimally invasive glucose sensor technology has been shown to improve glycaemic control in people living with T1D. We found clinically significant improvements in HbA1c levels in adult patients with T1D, that continued to improve between years one and two of sensor use, particularly in those with a pre‐sensor HbA1c of ≥64 mmol/mol [8.0%]. Despite the benefits of sensor use, many of our patients still face a challenge in achieving the international glycaemic control targets for time in range and HbA1c.

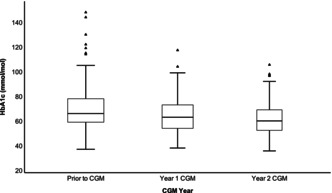

## 1 INTRODUCTION

Type 1 diabetes (T1D) is a chronic autoimmune disease that affects 8.4 million people worldwide, accounting for 5%–10% of all cases of diabetes.^1^, ^2^ The underlying pathophysiology of T1D involves the autoimmune destruction of insulin‐secreting pancreatic β‐cells, resulting in endogenous insulin deficiency and a lifelong dependence on exogenous insulin replacement therapy.^2^, ^3^ Maintaining stable glucose levels in T1D require continuous monitoring and insulin dose adjustments based on blood glucose levels.

Maintaining blood glucose levels in the normoglycaemic range has been shown to significantly reduce the risk of developing diabetes‐related complications.^4^, ^5^ However, achieving this remains challenging in T1D.^6^ There are several barriers to optimising glycaemia in T1D, including integrating diabetes management into day‐to‐day life, fear of hypoglycaemia, insulin dose calculation, as well as the inconvenience of frequent self‐monitoring of blood glucose (SMBG) levels.^7^ Recent advances in minimally invasive glucose sensor technology, which measure real‐time interstitial fluid glucose levels, have enabled people with T1D to monitor their glucose levels more easily and accurately. The use of this technology aids diabetes self‐management and has been shown to improve glycaemic metrics for patients using both multiple daily injections (MDI) of insulin and insulin pumps.^8^, ^9^, ^10^, ^11^, ^12^, ^13^

This study aims to evaluate the real‐world changes in glycated haemoglobin (HbA_1_c) and other glucose sensor‐based glycaemic metrics following the initiation of a glucose sensor in adults with T1D in routine clinical care.

## 2 METHODS

This was a retrospective, observational, single‐centre service study involving adult patients with T1D who use glucose sensors and attend a hospital‐based diabetes clinic in Dublin, Ireland. Data were collected from August 2021 to May 2022. As a service evaluation, no ethical approval was required. Patients had provided informed consent for their data to be remotely linked and shared with the diabetes clinic.

Data on gender, age, duration of diabetes, types of insulin therapy, HbA_1_c, duration of CGM use and the completion of the Dose Adjustment for Normal Eating (DAFNE) structured diabetes education course were collected. The manufacturers' proprietary web‐based glucose monitoring platforms, including Libreview (Abbott Diabetes Care; Oxon, UK), Dexcom Clarity (Dexcom Inc, San Diego, CA, USA) and Carelink (Medtronic Inc, MN, USA) were reviewed. Input on the glucose sensor type, percentage of time CGM data was captured by sensor, average glucose, glucose management indicator (GMI), coefficient of variation (CV), percentage time in very high range (>13.9 mmol/L), percentage time in high range (10.0–13.9 mmol/L), percentage time in range (3.9–10.0 mmol/L), percentage time in low range (3.0–3.9 mmol/L) and percentage time in very low range (<3.0 mmol/L) were obtained. Changes in HbA_1_c values before and during sensor use were analysed, along with sensor based glycaemic metrics in accordance with international consensus on GCM reporting guidelines.^14^

Statistical analysis was performed using IBM SPSS Statistics for Macintosh, Version 27 (IBM Corp., Armonk, N.Y., USA). Nonparametric tests were used for data that was not normally distributed. Wilcoxon signed‐rank test was used to compare the most recent HbA_1_c values to the pre‐sensor values in all patients, including subgroup analyses based on sensor type and baseline treatment modalities. Similar test was used to compare the changes in HbA_1_c within group of patients who had either a pre‐sensor HbA_1_c < 64 mmol/mol [8.0%] or ≥ 64 mmol/mol [8.0%]. To indicate the effect size, calculated *r* was performed with any value of above 0.1, 0.3 and 0.5 indicating small, medium and large effect, respectively. Friedman test was used to compare the changes in HbA_1_c pre‐sensor within the first and the second year on‐sensor. HbA_1_c within the first and second year on‐sensor was defined as the average HbA_1_c within that year. Data are presented as median (interquartile range) or mean ± standard deviation.

## 3 RESULTS

### 3.1 Baseline results

A summary of baseline characteristics of the patients (*n* = 209) for which the data was collected is presented in Table 1. Women accounted for 46.4% of patients. The median age, diabetes duration and duration of glucose sensor usage were 40 (30–53) years, 17 years (8.5–26) and 2.1 years (1.5–2.8), respectively. The majority of patients (93.3%) were using a real‐time continuous glucose monitor (RT‐CGM), while 6.7% were using a flash glucose monitor (FGM). Most patients (81.3%) were on MDI and 18.7% were using a continuous insulin pump (CSII) therapy. Within the RT‐CGM group, Dexcom G6 was the most popular option (*n* = 170), followed by Guardian 3 (*n* = 24) and Dexcom G5 (*n* = 1). A significant proportion of patients (66.5%) had completed the DAFNE structured diabetes education course. All patients received ongoing training in glucose sensor use, diabetes education and support during routine outpatient service appointments occurring approximately every 6 months.

**TABLE 1:** Patient characteristics.

Demographics	
Patients, *n*	209
Female, *n* (%)	97 (46.4%)
Age (years), median (IQR)	40 (30–53)
Diabetes duration (years), median (IQR)	17 (8.5–26)
Duration of CGM use (years), median (IQR)	2.09 (1.5–2.8)
Completed DAFNE, *n* (%)	139 (66.5%)

Diabetes treatment and monitoring	
Diabetes therapy, *n* (%)	
CSII	39 (18.7%)
MDI	170 (81.3%)
Glucose sensor, *n* (%)	
RT‐CGM	195 (93.3%)
Dexcom G5	1 (0.5%)
Dexcom G6	170 (81.3%)
Guardian 3	24 (11.5%)
FGM (Libre)	14 (6.7%)

Abbreviations: %, percentage; CSII, continuous subcutaneous insulin infusion; DAFNE, Dose Adjusted for Normal Eating; FGM, flash glucose monitor; IQR, interquartile range; MDI, multiple daily injection; n, number; RT‐CGM, real‐time continuous glucose monitoring.

The analysis of sensor‐based glycaemic control metrics is shown in Table 2. Overall, the median percentage of sensor use, and time spent in range (3.9–10.0 mmol/L) were 97% (89–99) and 48.4% (33–63), respectively. Overall, 16.7% of patients achieved a time in range ≥ 70% while 91.9% of patients met the international recommendation for time below range (<3.9 mmol/L) of less than 4%.

**TABLE 2:** Summary of sensor‐based metric results.

Glucose sensor metrics	*n*	Median (IQR)
% of time CGM data was captured by sensor	208	97 (88.9–99.0)
Average glucose levels (mmol/L)	208	10.4 (9.1–12.0)
GMI (mmol/mol)	171	61.7 (55.2–70.5)
CV %	203	32.5 (29.2–36.8)
% Time in very high range (>13.9 mmol/L)	208	16.4 (6.1–31.2)
% Time in high range (10.1–13.9 mmol/L)	208	29.4 (23.1–36.8)
% Time in range (3.9–10.0 mmol/L)	208	48.5 (33–63)
% Time in low range (3.0–3.8 mmol/L)	208	0.7 (0.1–1.4)
% Time in very low range (<3.0 mmol/L)	208	0.1 (0.0–0.3)

Abbreviations: %, percentage; CV, Coefficient of Variation; IQR, interquartile range; GMI, glucose management indicator; n, number.

### 3.2 HbA_1_c changes

**FIGURE 1:**
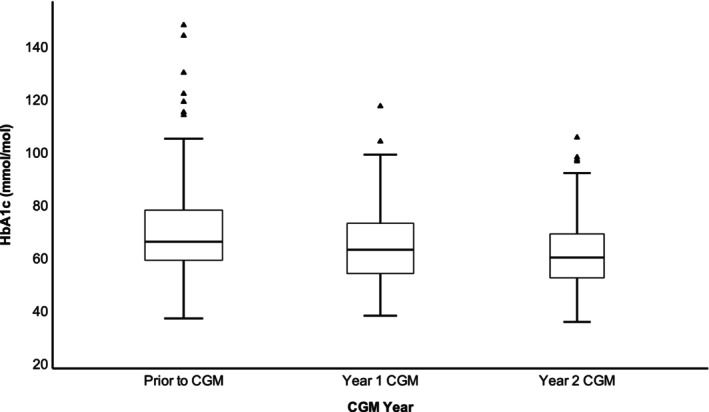
Box plot representing HbA_1_c change within 2 years of starting a glucose sensor; CGM, continuous glucose monitor; HbA_1_c, glycated haemoglobin.

Compared to the pre‐sensor HbA_1_c, there was a significant reduction in the most recent HbA_1_c at 60 (53–71) mmol/mol [7.6 (7.0–8.6)%] vs. 66 (59–78) mmol/mol [8.2 (7.5–9.3)%] (*p* < .001) (Table 3). Out of 209 patients, 57.9% (*n* = 121) were using glucose sensor for a minimum of 2 years. There was a significant improvement in HbA_1_c within the second year (60 (53–69) mmol/mol [7.6 (7.0–8.4)%]), compared to within the first year (62 (54–71) mmol/mol [7.8 (7.1–8.7)%]) and prior to the use of sensor (66 (57–74) mmol/mol [8.2 (7.4–8.9)%]) (*p* < .001) (Table 4 and Figure 1).

**TABLE 3:** Pre‐sensor HbA_1_c compared to the most recent HbA_1_c (*n* = 209).

Pre CGM HbA_1_c	Most recent HbA_1_c	*p* Value	Calculated *r*
66 (59–78) [8.2 (7.5–9.3)]	60 (53–71) [7.6 (7.0–8.6)]	<.001	0.35 (medium effect)

Abbreviations: CGM, continuous glucose monitor; HbA_1_c, glycated haemoglobin.

*Note*: HbA_1_c (mmol/mol) and [%] are expressed in median (IQR). Within‐person changes assessed by the Wilcoxon Signed Ranks Test.

**TABLE 4:** HbA_1_c change within 2 years of starting a glucose sensor (*n* = 121).

Pre CGM HbA_1_c	HbA_1_c within 1st year of CGM	HbA_1_c within 2nd year of CGM	*p* Value
66 (57–74) [8.2 (7.4–8.9)]	62 (54–71) [7.8 (7.1–8.7)]	60 (53–69) [7.6 (7.0–8.4)]	<.001

Abbreviations: CGM, continuous glucose monitor; HbA_1_c, glycated haemoglobin.

*Note*: HbA_1_c (mmol/mol) and [%] are expressed in median (IQR). HbA_1_c change assessed by Friedman Test.

### 3.3 HbA_1_c changes based on sensor type

In the Libre (FGM) group (*n* = 13), there was a nonsignificant reduction in the most recent HbA_1_c (73 (59–83) mmol/mol [8.8 (7.5–9.7)%]) compared to the pre‐sensor HbA_1_c (79 (65–100) mmol/mol [9.4 (8.1–11.3)%]) (*p* = .279). However, in the Dexcom G6 group, (*n* = 168 with pre‐sensor HbA_1_c data; *n* = 163 with most recent HbA_1_c data), there was a significant reduction in the most recent HbA_1_c (60 (52–70) mmol/mol [7.6 (6.9–8.6)%]) compared to pre‐sensor HbA_1_c (66 (59–78) mmol/mol [8.2 (7.5–9.3)%]) (*p* < .001, *r* = 0.33). Similarly, in the Guardian 3 group (*n* = 23), a significant reduction in the most recent HbA_1_c (59 (51–69) mmol/mol [7.5 (6.8–8.5)%]) was seen compared to pre‐sensor HbA_1_c (68 (61–81) mmol/mol [8.4 (7.7–9.6)%]) (*p* < .001, *r* = 0.59) (Table 5).

**TABLE 5:** Pre‐sensor HbA_1_c compared to the most recent HbA_1_c by sensor type.

CGM type	*n*	Pre CGM HbA_1_c	*n*	Most recent HbA_1_c	*p* Value	Calculated *r*
Libre	13	79 (65–100) [9.4 (8.1–11.3)]	13	73 (59–83) [8.8 (7.5–9.7)]	.279	–
Dexcom G6	168	66 (59–78) [8.2 (7.5–9.3)]	163	60 (52–70) [7.6 (6.9–8.6)]	<.001	0.33 (medium effect)
Dexcom G5	1	–		–	–	–
Guardian 3	23	68 (61–81) [8.4 (7.7–9.6)]	23	59 (51–69) [7.5 (6.8–8.5)]	<.001	0.59 (large effect)

Abbreviations: CGM, continuous glucose monitor; HbA_1_c, glycated haemoglobin; n, number.

*Note*: HbA_1_c (mmol/mol) and [%] are expressed in median (IQR). Within‐person changes assessed by the Wilcoxon Signed Ranks Test.

### 3.4 HbA_1_c changes based on baseline pre‐sensor HbA_1_c

A total of 38.3% of patients (*n* = 80) recorded a pre‐sensor HbA_1_c of <64 mmol/mol. In this group, there was a significant reduction in the most recent HbA_1_c (54 (49–59) mmol/mol [7.1 (6.6–7.5)%]) compared to the HbA_1_c prior to sensor (57 (52–61) mmol/mol [7.4 (6.9–7.7)%]) (*p* = .019, calculated *r* = 0.19) (Table 6).

**TABLE 6:** Patients with pre‐sensor HbA_1_c < 64 mmol/mol [8.0%] compared to most recent HbA_1_c.

	*n*	HbA_1_c (mmol/mol) [%]	*p* Value	Calculated r
Pre CGM	80	57 (52–61) [7.4 (6.9–7.7)]	.019	0.19 (small effect)
Most recent	78	54 (49–59) [7.1 (6.6–7.5)]		

Abbreviations: %, percentage; CGM, continuous glucose monitor; HbA_1_c, glycated haemoglobin; n, number.

*Note*: Assessed by Wilcoxon Signed Ranks Test.

A total of 59.8% of patients (*n* = 125) recorded a pre‐sensor HbA_1_c of ≥64 mmol/mol [8.0%]. In this group, there was a significant reduction in the most recent HbA_1_c (67 (59–75) mmol/mol [8.2 (7.6–9.0)%]) compared to the HbA_1_c prior to sensor (75 (68–83) mmol/mol [9.0 (8.4–9.7)%]) (*p* < .001, *r* = 0.44) (Table 7).

**TABLE 7:** Patients with pre‐sensor HbA_1_c ≥ 64 mmol/mol compared to most recent HbA_1_c.

	*n*	HbA_1_c (mmol/mol) (%)	*p* Value	Calculated *r*
Pre CGM	125	75 (68–83) [9.0 (8.4–9.7)]	<.001	0.44 (medium effect)
Most recent	120	67 (59–75) [8.2 (7.6–9.0)]		

Abbreviations: %, percentage; CGM, continuous glucose monitor; HbA_1_c, glycated haemoglobin; n, number.

*Note*: Assessed by Wilcoxon Signed Ranks Test.

### 3.5 HbA_1_c changes based on baseline diabetes treatment (MDI or CSII)

In the MDI group, (*n* = 167 with pre‐sensor HbA_1_c; *n* = 162 patients with most recent HbA_1_c), there was a significant reduction in the most recent HbA_1_c (60 (53–72) mmol/mol [7.6 (7.0–8.7)%]) compared to the pre‐sensor HbA_1_c (66 (59–78) mmol/mol [8.2 (7.5–9.3)%]) (*p* < .001, *r* = 0.33). Similarly, in the CSII group (*n* = 38), a significant reduction was seen in the most recent HbA_1_c (60 (53–68) mmol/mol [7.6 (7.0–8.4)%]) compared to the pre‐sensor HbA_1_c (67 (60–79) mmol/mol [8.3 (7.6–9.4)%]) (*p* < .001, *r* = 0.41) (Table 8).

**TABLE 8:** Pre‐sensor HbA_1_c compared to the most recent HbA_1_c by diabetes treatment type.

MDI or CSII	*n*	Pre CGM HbA_1_c	*n*	Most recent HbA_1_c	*p* Value	Calculated *r*
MDI	167	66 (59–78) [8.2 (7.5–9.3)]	162	60 (53–72) [7.6 (7.0–8.7)]	<.001	0.33 (medium effect)
CSII	38	67 (60–79) [8.3 (7.6–9.4)]	38	60 (53–68) [7.6 (7.0–8.4)]	<.001	0.41 (medium effect)

Abbreviations: CGM, continuous glucose monitor; CSII, continuous subcutaneous insulin infusion; HbA_1_c, glycated haemoglobin; MDI, multiple daily injection; n, number.

*Note*: HbA_1_c (mmol/mol) and [%] are expressed in median (IQR). Within‐person changes assessed by the Wilcoxon Signed Ranks Test.

## 4 DISCUSSION

In our study, we observed clinically significant improvements in HbA_1_c in adults with T1D using glucose sensors. Our real‐world results were similar to a large, retrospective, observational, single‐centre study involving 789 adults with T1D. In this study, HbA_1_c decreased from 61 (54–71) mmol/mol [7.7 (7.1–8.6)%] to 57 (49–66) mmol/mol [7.4 (6.6–8.2)%] in 561 participants using a flash glucose monitor (FGM), and from 60 (50–70) mmol/mol [7.6 (6.7–8.6)%] to 59 (50–67) mmol/mol [7.5 (6.8–8.3)%] in 198 participants using real‐time CGM (RT‐CGM) within 1 year of glucose sensor initiation.^9^ In another retrospective observational study (REAL‐CGM‐T1D study), real‐world glycaemic outcomes among 286 matched CGM naive adults with T1D who initiated a RT‐CGM were compared to FGM at 6 to 12 months follow up. In the RT‐CGM cohort (*n* = 143), HbA_1_c was reduced from a baseline of 68 ± 12 mmol/mol [8.4 ± 3.2%] to 60 ± 11 mmol/mol [7.6 ± 3.2%]. In the FGM cohort (*n* = 143), HbA_1_c was reduced from a baseline of 68 ± 11 mmol/mol [8.4 ± 3.2%] to 63 ± 12 mmol/mol [7.9 ± 3.2%].^15^ While we observed a similar reduction in HbA_1_c during the first year of sensor usage, our additional HbA_1_c data suggests that a continued reduction is observed in the second year of sensor use, highlighting the value in encouraging patients to continue using their sensor, even if they do not experience the predicted HbA_1_c reduction within their first year of sensor use.

Although both cohorts in the REAL‐CGM‐T1D study exhibited clinically significant reductions in HbA_1_c from baseline, the RT‐CGM cohort demonstrated a greater reduction in HbA_1_c of −3 mmol/mol [95% CI, −5 mmol/mol to −1 mmol/mol]; −0.3% [95% CI, −0.5% to −0.1%] (*p* = .01) compared to the FGM (FreeStyle Libre first‐generation) cohort.^15^ Similar findings were observed in the ALERTT1 trial. This was a 6‐month, multicentre, prospective, randomised controlled trial comparing RT‐CGM with FGM (FreeStyle Libre first‐generation) in 254 adults with T1D, previously using FGM. In this unselected adults T1D population, 6 months of RT‐CGM use led to an improved HbA_1_c from 57 (56 to 60) mmol/mol [7.4 (7.3 to 7.6)%] to 54 (52 to 55) mmol/mol [7.1 (6.9 to 7.2)%], while those remaining on FGM showed no improvement (mean difference − 4 mmol/mol [95% Cl, −5 mmol/mol to −3 mmol/mol]; −0.36% [−0.48% to −0.24%]) (*p* < .0001).^16^ Our study observed a significant improvement in HbA_1_c in the RT‐CGM groups (Dexcom G6 and Guardian 3) with medium to large effect size. However, such a significant improvement was not observed in the FGM group (Libre). This may be due to a smaller sample size (*n* = 13).

In a recent meta‐analysis, the HbA_1_c outcome was compared following intervention with CGM to SMBG from 22 studies involving participants with T1D. This included 2149 participants with a study duration ranged from <8 weeks (3 studies), 14–16 weeks (5 studies) and > 24 weeks (13 studies). The use of CGM significantly reduced the HbA_1_c levels compared with SMBG, with a mean difference of −2.46 mmol/mol [−0.23%]. Larger effects were observed among participants with higher baseline HbA_1_c > 64 mmol/mol [8.0%], with a mean difference of −4.67 mmol/mol [−0.43%].^8^ The tendency of individuals with T1D to maintain a high glucose levels in order to avoid hypoglycaemia, is more commonly observed in individuals with higher HbA_1_c.^17^ The use of a glucose sensor has been shown to reduce fear of hypoglycaemia,^18^ thus potentially contributing to the greater glycaemic benefits seen in participants with the higher baseline HbA_1_c compared to participants with lower HbA_1_c.

We observed a significant improvement in HbA1c in the MDI and CSII groups, with both exhibiting medium effect size. These findings suggest that the use of glucose sensor provides benefit to both groups. The minor difference in the effect size observed in the CSII group may be attributed to patients familiarity with diabetes technology, the ability to administer a more precise insulin adjustment and utilising sensor‐augmented pump therapy with predictive low glucose suspend capabilities including other advanced hybrid closed loop features.^19^, ^20^

Despite observing significant improvements in HbA_1_c levels over time with the introduction of glucose sensor technology, achieving the recommended goals for all glycaemic metrics, as defined by the ADA standards of care (2021),^21^ remained challenging. In our cohort, 25.8% of patients achieved the recommended HbA_1_c goal of ≤53 mmol/mol [7.0%], while 16.7% achieved the recommended TIR of ≥70% and 91.9% achieved the recommended goal of <4% for time below range. In a multinational cohort study including 5219 children, adolescents, and young adults with T1D, the proportion of individuals achieving the recommended time in range target was found to be associated with treatment modality. Users of RT‐CGM concurrently with an insulin pump were the most likely to achieve >70% time in range.^22^ In recent years, we have seen a widespread use of advanced hybrid closed loop (ACHL) technology that demonstrates real‐world success in safely achieving these glycaemic targets,^23^, ^24^, ^25^, ^26^ and is a potential tool to further improve the HbA_1_c levels. This trend suggests that the use of insulin pumps and closed loop technologies with glucose sensors may be required, for the more precise glycaemic control required to achieve recommended clinical targets.

The strengths of this study included the real‐world nature of the results, the sample size, use of average HbA_1_c values to assess changes during the first and the second year of sensor use, and a high level of sensor data availability. There were several limitations to our study. This was a retrospective observational study evaluating the impact of introducing a glucose sensor, which was limited to Dexcom, Guardian 3 and Libre 1, in unselected patients with T1D attending a diabetes service in a public hospital. Furthermore, patients' options were impacted by the CGM funding at the time in which Libre 1 was approved to patients under 21 years old while Dexcom and Guardian 3 were approved for all ages. Consequently, the cohort of patients that we identified may have affected the data. Additionally, there are several factors that may contribute to HbA_1_c changes such as diabetes severity index, the presence of diabetes related complications, duration of diabetes, age at diagnosis, rate of DAFNE completion, patients receiving other adjunctive noninsulin therapies, outpatient review frequency and nonattendance rate.^27^, ^28^ These factors may need to be controlled in future studies. A small number of patients were using sensor‐augmented pump therapy with predictive low glucose suspend capabilities (Medtronic 640G) (*n* = 12) and advanced hybrid closed loop system (Medtronic 780G) (*n* = 12). The frequency of attendance for laboratory HbA_1_c measurements may also have been reduced due to the COVID‐19 pandemic. Both factors may contribute to the changes in HbA_1_c seen in this study.

## 5 CONCLUSION

We observed a clinically significant and sustained improvements in HbA_1_c levels in adult patients with T1D during the first 2 years of glucose sensor use. This suggests a potential benefit of employing long‐term CGM usage in our clinical practice, mainly in patients with HbA_1_c ≥ 64 mmol/mol, either on MDI or CSII. Despite the benefits of sensor use, most patients still face challenges in achieving the international glycaemic targets of >70% TIR (3.9–10.0 mmol/L) and HbA_1_c < 53 mmol/mol [7.0%]. Our study highlights the need for continuous development of sensor technology, its availability, and the ongoing education and support for patients and diabetes educators in optimising glycaemic metrics.

## AUTHOR CONTRIBUTIONS

**Robert E. Lyons:** Conceptualization (supporting); data curation (lead); formal analysis (equal); investigation (supporting); methodology (supporting); project administration (lead); writing – original draft (lead); writing – review and editing (lead). **Roshaida Abdul Wahab:** Data curation (equal); formal analysis (lead); methodology (equal); supervision (equal); writing – review and editing (lead). **Sue Yee Goh:** Data curation (lead); methodology (equal); project administration (equal); writing – review and editing (supporting). **Cathy Breen:** Conceptualization (equal); data curation (equal); writing – review and editing (equal). **Amanda Rhynehart:** Conceptualization (equal); data curation (equal); writing – review and editing (supporting). **Mary O'Scannail:** Conceptualization (equal); data curation (equal); writing – review and editing (supporting). **Hannah Jade Kelly:** Conceptualization (equal); data curation (equal); writing – review and editing (supporting). **Karl Neff:** Conceptualization (equal); data curation (equal); writing – review and editing (supporting). **Donal O'Shea:** Conceptualization (equal); data curation (equal); writing – review and editing (supporting). **Ronan Canavan:** Conceptualization (equal); data curation (equal); writing – review and editing (supporting). **Wan Aizad Wan Mahmood:** Conceptualization (lead); data curation (supporting); formal analysis (supporting); funding acquisition (lead); investigation (lead); methodology (lead); project administration (lead); resources (lead); software (lead); supervision (lead); validation (lead); visualization (lead); writing – original draft (supporting); writing – review and editing (lead).

## FUNDING INFORMATION

The author(s) reported there is no funding associated with the work featured in this article.

## CONFLICT OF INTEREST STATEMENT

REL, RAW, SYG, AR, MOS, HJK, KN, DOS, RC and WAWM declare no conflict of interest. CB declares receiving honoraria for educational events and conference attendance from Astra Zeneca, Behaviour Change Training Ltd., Diabetes Ireland, EASO, International Medical Press, Eli Lily, Medscape, MSD, Novo Nordisk and Sanofi Aventis and is a former member of a Dexcom Advisory Board. She is a member of an Obesity National Clinical Programme Clinical Advisory Group, and MECC working group in Ireland.

## Data Availability

Data sharing is not applicable to this article as no new data were created or analyzed in this study.

## References

[1] Gregory GA , Robinson TIG , Linklater SE , et al. Global incidence, prevalence, and mortality of type 1 diabetes in 2021 with projection to 2040: a modelling study. Lancet Diabetes Endocrinol. 2022;10(10):741‐760. 36113507 10.1016/S2213-8587(22)00218-2

[2] Committee ADAPP . 2. Classification and diagnosis of diabetes: standards of medical Care in Diabetes—2022. Diabetes Care. 2021;45(Supplement_1):S17‐S38. 10.2337/dc22-S002 34964875

[3] Atkinson MA , Eisenbarth GS , Michels AW . Type 1 diabetes. The Lancet. 2014;383(9911):69‐82. 10.1016/S0140-6736(13)60591-7 PMC4380133 23890997

[4] Nathan DM , Genuth S , Lachin J , et al. The effect of intensive treatment of diabetes on the development and progression of long‐term complications in insulin‐dependent diabetes mellitus. N Engl J Med. 1993;329(14):977‐986. 8366922 10.1056/NEJM199309303291401

[5] Nathan DM , Cleary PA , Backlund JY , et al. Intensive diabetes treatment and cardiovascular disease in patients with type 1 diabetes. N Engl J Med. 2005;353(25):2643‐2653. 16371630 10.1056/NEJMoa052187 PMC2637991

[6] Prigge R , McKnight JA , Wild SH , et al. International comparison of glycaemic control in people with type 1 diabetes: an update and extension. Diabet Med. 2022;39(5):e14766. 34890078 10.1111/dme.14766

[7] Vincze G , Barner JC , Lopez D . Factors associated with adherence to self‐monitoring of blood glucose among persons with diabetes. Diabetes Educ. 2004;30(1):112‐125. 14999899 10.1177/014572170403000119

[8] Teo E , Hassan N , Tam W , Koh S . Effectiveness of continuous glucose monitoring in maintaining glycaemic control among people with type 1 diabetes mellitus: a systematic review of randomised controlled trials and meta‐analysis. Diabetologia. 2022;65(4):604‐619. 35141761 10.1007/s00125-021-05648-4

[9] Lee K , Gunasinghe S , Chapman A , et al. Real‐world outcomes of glucose sensor use in type 1 diabetes‐findings from a large UK Centre. Biosensors (Basel). 2021;11(11):457. 34821673 10.3390/bios11110457 PMC8615559

[10] Mulinacci G , Alonso GT , Snell‐Bergeon JK , Shah VN . Glycemic outcomes with early initiation of continuous glucose monitoring system in recently diagnosed patients with type 1 diabetes. Diabetes Technol Ther. 2019;21(1):6‐10. 30575413 10.1089/dia.2018.0257

[11] Nathanson D , Svensson AM , Miftaraj M , Franzén S , Bolinder J , Eeg‐Olofsson K . Effect of flash glucose monitoring in adults with type 1 diabetes: a nationwide, longitudinal observational study of 14,372 flash users compared with 7691 glucose sensor naive controls. Diabetologia. 2021;64(7):1595‐1603. 33774713 10.1007/s00125-021-05437-z PMC8187189

[12] Tyndall V , Stimson RH , Zammitt NN , et al. Marked improvement in HbA(1c) following commencement of flash glucose monitoring in people with type 1 diabetes. Diabetologia. 2019;62(8):1349‐1356. 31177314 10.1007/s00125-019-4894-1 PMC6647076

[13] Deshmukh H , Wilmot EG , Gregory R , et al. Effect of flash glucose monitoring on glycemic control, hypoglycemia, diabetes‐related distress, and resource utilization in the Association of British Clinical Diabetologists (ABCD) Nationwide audit. Diabetes Care. 2020;43(9):2153‐2160. 32669277 10.2337/dc20-0738 PMC7440900

[14] Battelino T , Danne T , Bergenstal RM , et al. Clinical targets for continuous glucose monitoring data interpretation: recommendations from the international consensus on time in range. Diabetes Care. 2019;42(8):1593‐1603. 31177185 10.2337/dci19-0028 PMC6973648

[15] Brown RE , Chu L , Norman GJ , Abitbol A . Real‐world glycaemic outcomes in adult persons with type 1 diabetes using a real‐time continuous glucose monitor compared to an intermittently scanned glucose monitor: a retrospective observational study from the Canadian LMC diabetes registry (REAL‐CGM‐T1D). Diabet Med. 2022;39(11):e14937. 36065977 10.1111/dme.14937 PMC9826315

[16] Visser MM , Charleer S , Fieuws S , et al. Comparing real‐time and intermittently scanned continuous glucose monitoring in adults with type 1 diabetes (ALERTT1): a 6‐month, prospective, multicentre, randomised controlled trial. Lancet. 2021;397(10291):2275‐2283. 34089660 10.1016/S0140-6736(21)00789-3

[17] Blauw H , Keith‐Hynes P , Koops R , DeVries JH . A review of safety and design requirements of the artificial pancreas. Ann Biomed Eng. 2016;44(11):3158‐3172. 27352278 10.1007/s10439-016-1679-2 PMC5093196

[18] Beck RW , Lawrence JM , Laffel L , et al. Quality‐of‐life measures in children and adults with type 1 diabetes: Juvenile Diabetes Research Foundation continuous glucose monitoring randomized trial. Diabetes Care. 2010;33(10):2175‐2177. 20696865 10.2337/dc10-0331 PMC2945155

[19] Jendle JH , Rawshani A , Svensson AM , Avdic T , Gudbjörnsdóttir S . Indications for insulin pump therapy in type 1 diabetes and associations with glycemic control. J Diabetes Sci Technol. 2016;10(5):1027‐1033. 27226388 10.1177/1932296816650209 PMC5032960

[20] Weisman A , Bai JW , Cardinez M , Kramer CK , Perkins BA . Effect of artificial pancreas systems on glycaemic control in patients with type 1 diabetes: a systematic review and meta‐analysis of outpatient randomised controlled trials. Lancet Diabetes Endocrinol. 2017;5(7):501‐512. 28533136 10.1016/S2213-8587(17)30167-5

[21] American Diabetes Association . 6. Glycemic targets: standards of medical care in diabetes‐2021. Diabetes Care. 2021;44(Suppl 1):S73‐s84. 33298417 10.2337/dc21-S006

[22] Dovc K , Lanzinger S , Cardona‐Hernandez R , et al. Association of achieving time in range clinical targets with treatment modality among youths with type 1 diabetes. JAMA Netw Open. 2023;6(2):e230077. 36808243 10.1001/jamanetworkopen.2023.0077 PMC9941889

[23] Silva JD , Lepore G , Battelino T , et al. Real‐world performance of the MiniMed™ 780G system: first report of outcomes from 4120 users. Diabetes Technol Ther. 2022;24(2):113‐119. 34524003 10.1089/dia.2021.0203 PMC8817690

[24] Pinsker JE , Müller L , Constantin A , et al. Real‐world patient‐reported outcomes and glycemic results with initiation of control‐IQ technology. Diabetes Technol Ther. 2021;23(2):120‐127. 32846114 10.1089/dia.2020.0388 PMC7868573

[25] Peacock S , Frizelle I , Hussain S . A systematic review of commercial hybrid closed‐loop automated insulin delivery systems. Diabetes Ther. 2023;14(5):839‐855. 37017916 10.1007/s13300-023-01394-5 PMC10126177

[26] Isganaitis E , Raghinaru D , Ambler‐Osborn L , et al. Closed‐loop insulin therapy improves glycemic control in adolescents and young adults: outcomes from the international diabetes closed‐loop trial. Diabetes Technol Ther. 2021;23(5):342‐349. 33216667 10.1089/dia.2020.0572 PMC8080922

[27] ElSayed NA , Aleppo G , Aroda VR , et al. 7. Diabetes technology: standards of care in diabetes‐2023. Diabetes Care. 2023;46(Suppl 1):S111‐S127. 36507635 10.2337/dc23-S007 PMC9810474

[28] Glasheen WP , Renda A , Dong Y . Diabetes complications severity index (DCSI)‐update and ICD‐10 translation. J Diabetes Complications. 2017;31(6):1007‐1013. 28416120 10.1016/j.jdiacomp.2017.02.018

